# Decoupled  Anti-Diabetic and Gut Microbiota Responses to *Lactiplantibacillus plantarum* LPJZ-658 in db/db Mice

**DOI:** 10.3390/microorganisms14102361

**Published:** 2026-10-15

**Authors:** Cuiqing Zhao, Huayi Lu, Fangbing Liu, Yuxin Qiao

**Affiliations:** 1 Department of Pharmacy, Beihua University, Jilin 132013, China; 2 Department of Ophthalmology, The First Affiliated Hospital of USTC, Division of Life Sciences and Medicine, University of Science and Technology of China, Hefei 230001, China

**Keywords:** *Lactiplantibacillus plantarum* LPJZ-658, sex dependent, type 2 diabetes mellitus, anti-diabetic, gut microbiota

## Abstract

This study was conducted to investigate the sex-dependent effect of *Lactiplantibacillus plantarum* LPJZ-658 on anti-diabetic efficacy and gut microbiota modulation in db/db mice. Eight-week-old male and female db/db diabetic mice were administered LPJZ-658 (1 × 10^9^ CFU/day) or PBS for 4 weeks, with db/m mice as the healthy controls. Body weight, food intake, and fasting serum glucose were measured weekly. After intervention, serum levels of glucose, insulin, and lipid profile parameters (TG, T-CHO, NEFA, HDL, and LDL), as well as liver enzymes (AST and ALT), were determined. Histopathological changes in the kidney, pancreas, and liver were assessed, and the gut microbiota were analyzed. For most metabolic and histological parameters, the sex × treatment interaction was not significant. Nevertheless, post hoc multiple comparisons demonstrated that LPJZ-658 exerted marked anti-diabetic benefits predominantly in male db/db mice. In male db/db mice, LPJZ-658 reduced food intake, relative body weight, liver/body weight ratio, and serum glucose, insulin, TG, and ALT levels. Additionally, it alleviated histopathological damage to the kidney, pancreas, and liver. In contrast, female db/db mice showed no significant improvements in metabolic or histological parameters following LPJZ-658 treatment. Notably, gut microbiota indicators, including α-diversity and phylum-level and genus-level bacterial relative abundances exhibited significant sex × treatment interactions. Stratified analyses revealed that LPJZ-658 treatment showed a trend toward shifting the overall community structure toward the db/m phenotype and modulated multiple taxa at the phylum and genus levels in male db/db mice, whereas the microbial community structure and α-diversity remained largely unchanged in females. Additionally, functional prediction indicated that LPJZ-658 modulated gut microbial metabolic pathways in male mice, including membrane transport, carbohydrate metabolism, and amino acid metabolism pathways. LPJZ-658 exhibits sex-dependent anti-diabetic effects, with significant benefits likely associated with sex-specific modulation of the gut microbiota observed predominantly in males. Therefore, sex should be considered a critical factor in probiotic interventions for type 2 diabetes.

## 1. Introduction

Diabetes mellitus (DM) is a global health issue that affects people of all ages and genders, and its prevalence is rising rapidly worldwide, especially in developing countries [1]. It is estimated that in 2024, 589 million adults were living with DM, and this number is projected to rise to 853 million by 2050 [2]. DM is a metabolic disease that can dysregulate carbohydrate, fat, and protein metabolism, and it is associated with a cascade of macrovascular (such as cardiovascular disease) and microvascular complications (such as nephropathy, retinopathy, and neuropathy) that significantly reduce quality of life and increase mortality rates [3,4]. It is worth noting that a growing number of epidemiological and experimental studies indicate significant sex differences in the pathogenesis, progression, and clinical outcomes of DM and its complications [5,6]. Clinically, male patients with DM are more prone to insulin resistance, visceral obesity, peripheral neuropathy, and diabetic nephropathy, whereas female DM patients exhibit a higher risk of cardiovascular complications [7,8,9]. These sex differences emerge from complex interactions among biological factors (such as sex hormones and chromosomal differences), lifestyle factors (such as diet and physical activity), and gut microbiota composition [10], highlighting the need for sex-specific therapeutic strategies.

Probiotics are defined as “live microorganisms that, when administered in adequate amounts, confer a health benefit on the host” [11] and have gained considerable attention as a safe and effective adjunct therapy for type 2 diabetes (T2DM). Among probiotic strains, *Lactiplantibacillus plantarum* (*L. plantarum*) is widely studied due to its ability to survive gastrointestinal transit, colonize the gut, and modulate metabolic pathways [12,13]. Previous studies have demonstrated that *L. plantarum* strains improve glucose homeostasis, reduce lipid accumulation, and alleviate organ damage in DM models by regulating gut microbiota composition, enhancing short-chain fatty acid (SCFA) production, and suppressing systemic inflammation [14,15,16]. *L. plantarum* ZJUFB2 has been shown to reduce blood glucose levels and improve insulin sensitivity in high-fat diet (HFD)-induced insulin-resistant mice by decreasing the abundance of obesity-related and inflammation-associated microbes and increasing that of SCFA and bile salt hydrolase-producing bacteria [17]. Similarly, *L. plantarum* HAC01 alleviates T2DM in mice by regulating gut microbiota-derived metabolites such as butyrate [16]. Additionally, *L. Plantarum* 84-3 alleviated T2DM-related glucose homeostasis by increasing circulating l-glutamine levels [18]. However, most existing probiotic studies on diabetes have focused on single-sex animal models, leading to a critical gap in our understanding of sex-dependent probiotic efficacy. However, recent research has indicated sex differences in the alleviating effects of probiotics on high-fat diet-induced hyperinsulinemia in mice [19]. This highlights the urgent need to investigate differences in the response to probiotic therapy among different sex populations to optimize its clinical application [20].

Accumulating evidence supports the sex-specific effects of probiotics on metabolic outcomes. A recent study demonstrated that *L. plantarum* AS21 significantly ameliorated glucose intolerance in naturally aged male mice while exerting minimal effects on metabolically stable females, an effect associated with sex-specific remodeling of mucosa-associated microbial communities [21]. Similarly, the abundance of Lactobacillus was substantially lower in male mice compared to female mice across all intestinal locations in a high-fat diet-induced T2DM model, and this genus showed marked sexual dimorphism [22]. Furthermore, sex steroid hormones, particularly estrogen and testosterone, play key roles in modulating gut microbiota composition and host metabolism [23]. These findings highlight the urgent need to investigate sex differences in the response to probiotic therapy to optimize its clinical application.

LPJZ-658 is a novel *L. plantarum* strain isolated by our research group at Jilin Agricultural Science and Technology University from naturally fermented dairy products. Our previous studies confirmed that LPJZ-658 displayed excellent probiotic properties, including high tolerance to gastric acid and bile salts, strong adhesion to intestinal epithelial cells, and excellent antimicrobial activity in vitro [24]. Furthermore, we found that LPJZ-658 treatment significantly alleviated liver injury in both non-alcoholic steatohepatitis (NASH) and metabolic dysfunction-associated steatotic liver disease (MASLD) mice, regulated serum bile acid levels, and improved gut microbiota homeostasis, further confirming its potential as a therapeutic probiotic [25,26]. However, the underlying mechanisms involving gut microbiota remodeling, as well as whether LPJZ-658 exhibits anti-diabetic effects, remain unclear. In this study, we utilized male and female db/db mice, a well-established T2DM model, to systematically evaluate the sex-specific efficacy of LPJZ-658. We measured key physiological parameters (body weight and food intake) and metabolic indicators (serum glucose, insulin, and lipid profile) and examined histopathological changes in target organs (kidney, pancreas, and liver). Additionally, we analyzed gut microbiota composition via 16S rRNA gene sequencing and predicted functional metabolic pathways using PICRUSt and KEGG analyses. We specifically tested for sex × treatment interactions in all parameters and, where significant, performed stratified analyses to identify sex-specific effects. The objectives of this study were to determine the sex-dependent effects of LPJZ-658 on anti-diabetic activities in db/db mice and to provide experimental evidence for the development of personalized probiotic therapies for T2DM.

## 2. Materials and Methods

### 2.1. Culture and Preparation of Probiotic LPJZ-658

*L. plantarum* LPJZ-658 was isolated from naturally fermented dairy products in Xinjiang, China [24]. This strain was initially maintained at Jilin Agricultural Science and Technology University by our research group. Currently, a portion of the preserved strain is stored at the laboratory at Beihua University, where it is being stably cultured and routinely subcultured. The strain was cultured in MRS broth (Beijing Solarbio Science & Technology Co., Ltd., Beijing, China) at 37 °C for 18–20 h. Cultured cells were harvested by centrifugation at 3000× *g* for 20 min, washed twice with physiological saline (PBS, Beijing Solebao Technology Co., Ltd., Beijing, China), and then re-suspended in PBS to a final concentration of 1 × 10^10^ CFU/mL.

### 2.2. Animals and Experimental Design

Eight-week-old male and female db/db mice (diabetic model) and C57BL/Ks mice (control, db/m) were purchased from Jilin Genet-Med Biotechnology Co., Ltd. (Jilin, China). This study was approved by the Beihua University Laboratory Animal Care and Use Committee. During the experiment, a normal chow diet and water were provided ad libitum. After a 1-week acclimation period, the mice were randomly divided into six groups (*n* = 7 for each group), as shown in Table 1. The control (db/m♂ and db/m♀) and diabetic model groups (db/db♂ and db/db♀) received daily intragastric (i.g.) administration of PBS for 4 weeks. In contrast, the LPJZ-658 treatment groups (db/db♂ + LPJZ-658 and db/db♀ + LPJZ-658) received daily i.g. administration of LPJZ-658 at a dosage of 1 × 10^9^ CFU/100 μL for 4 weeks. During the experimental period, body weight, food intake, and fasting serum glucose levels were monitored weekly. At the end of the intervention, mice under Avertin (2,2,2-tribromoethanol, Sigma-Aldrich, St Louis, MN, USA) anesthesia (250mg/kg i.p.) were euthanized via cervical dislocation. Blood samples were collected, and the liver, epididymal white adipose tissue (eWAT, male mice), and gonadal white adipose tissue (gWAT, female mice) were harvested, weighed, and recorded. Additionally, tissues of the liver, pancreas, and kidney were collected for histopathological analysis, while cecal contents were collected and stored at −80 °C for intestinal microbiota analysis.

### 2.3. Serum Biochemical Analysis

Serum levels of glucose, insulin, triglyceride (TG), total cholesterol (T-CHO), high-density lipoprotein cholesterol (HDL-C), low-density lipoprotein cholesterol (LDL-C), non-esterified fatty acid (NEFA), aspartate aminotransferase (AST), and alanine aminotransferase (ALT) were measured according to the instructions of the corresponding commercial enzyme-linked immunosorbent assay (ELISA) kits (Nanjing Jiancheng Biology Co., Ltd., Nanjing, China).

### 2.4. Histopathological Analysis

Liver, pancreas, and kidney tissues were fixed in 10% neutral-buffered formalin for 72 h, dehydrated through a graded ethanol series, and embedded in paraffin. Sections (5 μm thick) were made from each tissue; hematoxylin and eosin (H&E) staining was performed for histological evaluation, and pathological changes were recorded. Histopathological evaluation was performed in a blinded manner by two independent investigators.

### 2.5. Intestinal Microbiota Analysis

Cecal content samples were sent to Novogene Co., Ltd. (Beijing, China) for 16S rRNA gene sequencing. The V3-V4 hypervariable regions of the 16S rRNA gene were amplified, and sequencing was performed on the Illumina NovaSeq platform. Raw sequencing data were processed and analyzed using QIIME2 software(version 2026.7) to characterize the microbiota profiles, and additional bioinformatics analyses were conducted on the NovaMagic cloud platform (https://magic.novogene.com/ accessed on 28 September 2026).

### 2.6. Statistical Analysis

The data were processed using GraphPad Prism 8.0 (GraphPad Software, Inc., La Jolla, CA, USA). For normally distributed data, the mean ± SEM was used to represent the results. Two-way ANOVA was routinely applied to evaluate the main effects of sex and treatment, as well as their interaction (sex × treatment). For metabolic and histological parameters with non-significant sex × treatment interactions, only the main effects of sex and treatment were interpreted. For the repeated-measures data (body weight and food intake), a two-way repeated-measures ANOVA was used, followed by Tukey’s post hoc test. Gut microbiota analysis were performed using the Novomagic cloud platform (Novogene, Beijing, China). As described above, α-diversity indices and taxonomic abundances at the phylum and genus levels were analyzed using a two-way ANOVA. When a significant sex × treatment interaction was observed, data were stratified by sex and analyzed by one-way ANOVA followed by Tukey’s post hoc test within each sex. In contrast, β-diversity was assessed using principal coordinates analysis (PCoA) based on Bray–Curtis distances. Because the sex × treatment interaction was significant in univariate analyses, PERMANOVA (adonis) was performed separately for male and female mice to test the overall effect of treatment within each sex. The R^2^ and adjusted *p*-values from the pairwise PERMANOVA were annotated on the PCoA plots, and LEfSe was performed separately for male and female mice to identify differentially enriched taxa (LDA score > 4.0). KEGG functional pathways at level 2 were predicted using PICRUSt, and differences were considered statistically significant at *p* < 0.05.

## 3. Results

### 3.1. Effects of LPJZ-658 on General Physiological Parameters

**Figure 1:**
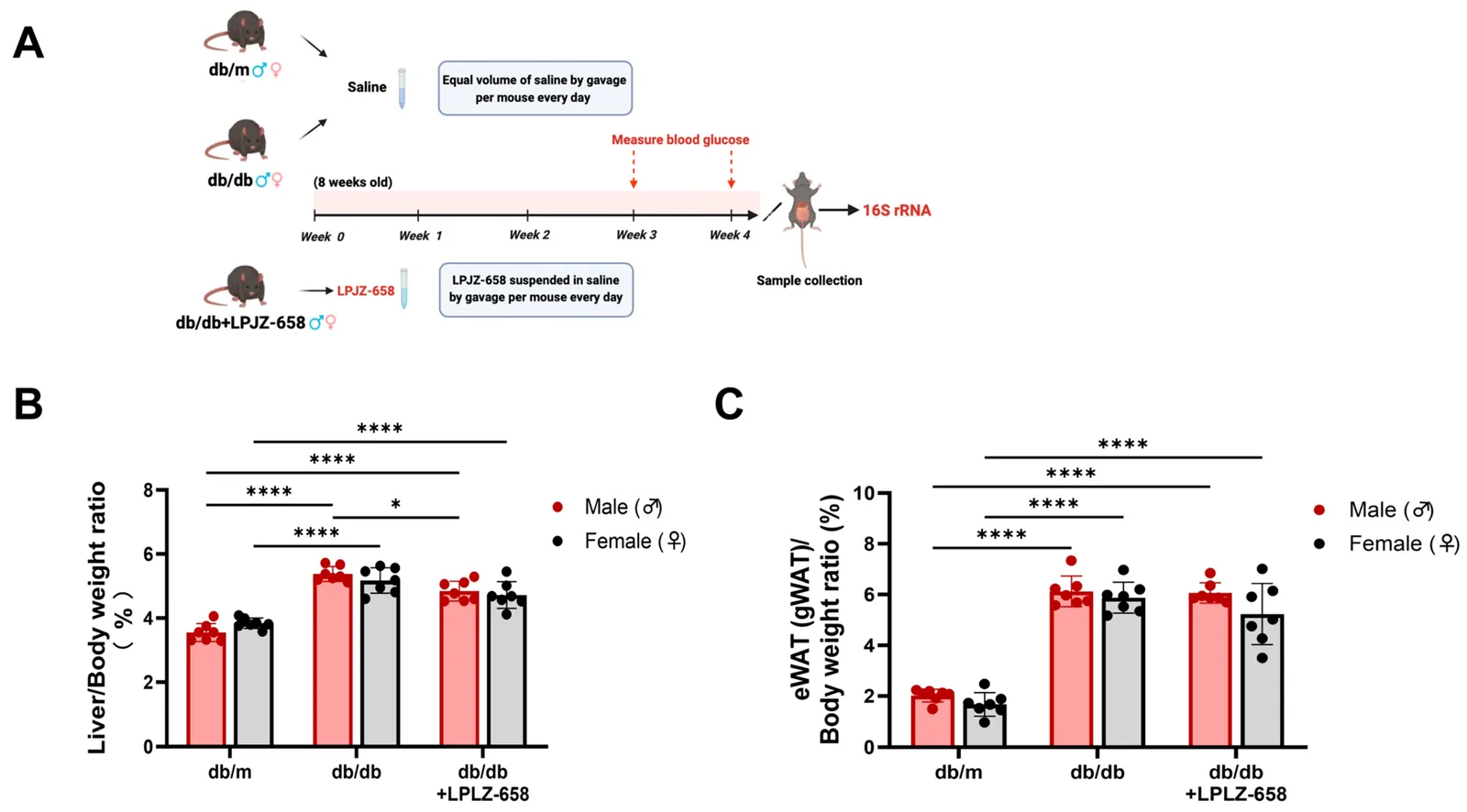
LPJZ-658 improves general physiological parameters in a sex-dependent manner. (**A**) Schematic diagram of the experimental design. (**B**) Liver/body weight ratio. (**C**) White adipose tissue/body weight ratio, including epididymal white adipose tissue (eWAT) for male mice and gonadal white adipose tissue (gWAT) for female mice. All data are presented as mean ± SEM (*n* = 7 for each group); * *p* < 0.05 and **** *p* < 0.0001. ♂, male; ♀, female.

To explore the sex-dependent anti-diabetic effects of LPJZ-658, male and female db/db mice were used to establish DM animal models. The experimental design is illustrated in Figure 1A. After 4 weeks of treatment, anti-diabetic efficacy was determined by measuring body weight, serum glucose, insulin, and lipid profile parameters. Additionally, pathological changes in the kidneys, pancreas, and liver were examined, and gut microbiota composition was analyzed.

As shown in Table 2, both male and female db/db mice exhibited significantly higher food intake than sex-matched db/m mice. Notably, female db/db mice had significantly lower food intake than male db/db mice. LPJZ-658 treatment significantly reduced food intake in male db/db mice throughout the 4-week period, whereas in female db/db mice, a significant reduction in food intake by LPJZ-658 was only observed at Weeks 2 and 4. Moreover, male db/db + LPJZ-658 mice displayed significantly lower food intake than female db/db + LPJZ-658 mice at Weeks 3 and 4.

As presented in Table 3, the relative body weight ratio of both male and female db/db mice was significantly higher than that of db/m mice from Week 2 onward. LPJZ-658 treatment consistently decreased the relative body weight ratio of male db/db mice beginning at Week 1, but this inhibitory effect was not observed in female db/db mice. Notably, there was no significant difference in the relative body weight ratio between male and female db/db mice. In addition, there was no significant difference in the relative body weight ratio between male and female db/db + LPJZ-658 mice except for Week 1. As shown in Figure 1B,C, both male and female db/db mice had significantly higher liver/body weight ratios and epididymal white adipose tissue (eWAT)/body weight ratios (or gWAT/body weight ratios) than db/m mice. Interestingly, after 4 weeks of LPJZ-658 treatment, the liver/body weight ratio was significantly reduced exclusively in male db/db mice. For these parameters, the sex × treatment interaction was not significant. However, the main effect of LPJZ-658 treatment was significant for the liver/body weight ratio, and the main effects of LPJZ-658 treatment and sex were significant for the eWAT/gWAT ratio.

### 3.2. Effect of LPJZ-658 on Serum Glucose, Serum Insulin, and Kidney/Pancreas Pathological Alterations

**Figure 2:**
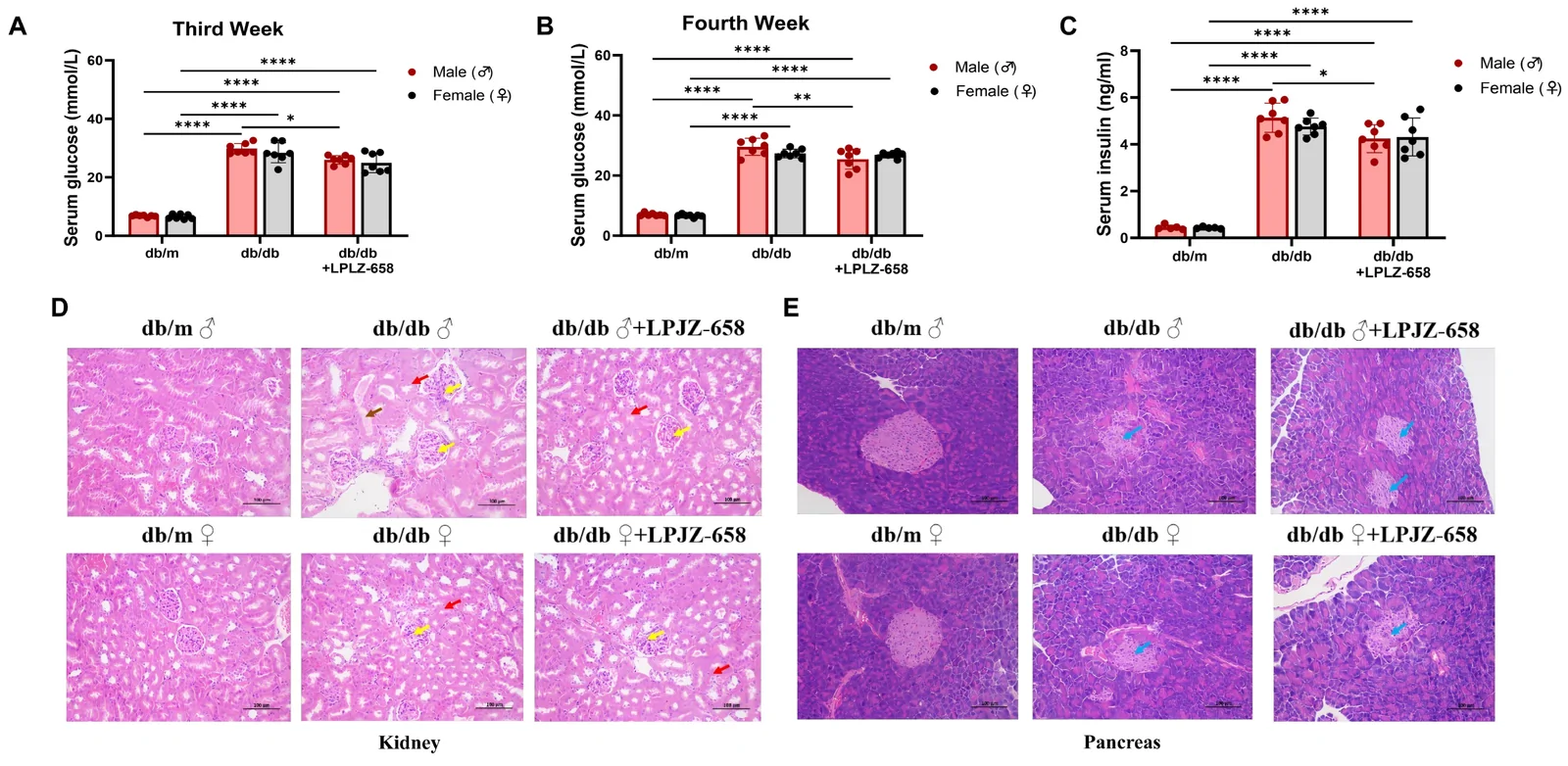
LPJZ-658 improves glucose homeostasis and attenuates renal and pancreatic histological damage in male db/db mice. (**A**) Serum glucose levels at the third week and (**B**) fourth week. (**C**) Serum insulin levels. (**D**) Representative hematoxylin and eosin (H&E) staining of kidney tissues and (**E**) pancreatic tissues. All data are presented as the mean ± SEM (*n* = 7 for each group); * *p* < 0.05, ** *p* < 0.01, and **** *p* < 0.0001. ♂, male; ♀, female.

To assess the impact of LPJZ-658 on glucose homeostasis, serum glucose levels were measured at the third and fourth weeks post-treatment. As shown in Figure 2A,B, both male and female db/db mice exhibited significantly elevated serum glucose levels compared with db/m mice. Notably, LPJZ-658 treatment significantly lowered serum glucose levels in male db/db mice but not in females. Consistently, serum insulin levels were markedly higher in both male and female db/db mice relative to db/m mice. Additionally, LPJZ-658 treatment significantly reduced serum insulin levels in male db/db mice only (Figure 2C). For serum glucose and insulin levels, the sex × treatment interaction was not significant, while the main effect of LPJZ-658 treatment was significant.

To assess the effect of LPJZ-658 on kidney injury in db/db mice, H&E staining was performed. As shown in Figure 2D, male db/db mice exhibited obvious kidney pathological changes: glomerular mesangial cell proliferation, basement membrane thickening (yellow arrows), renal tubular granular degeneration with loose cytoplasm and eosinophilic fine granules (red arrows), and eosinophilic deposits in a small number of renal tubules (brown arrows). After 4 weeks of LPJZ-658 treatment, male db/db + LPJZ-658 mice presented only mild glomerular mesangial proliferation (yellow arrows) and mild renal tubular granular degeneration (red arrows), indicating a significant alleviation of renal pathological lesions compared with male db/db mice. In contrast, H&E staining revealed only mild renal pathological changes in female db/db mice, including glomerular mesangial proliferation (yellow arrows) and mild granular degeneration in a few renal tubules (red arrows), and LPJZ-658 treatment did not induce a significant improvement in these pathological changes.

**Figure 3:**
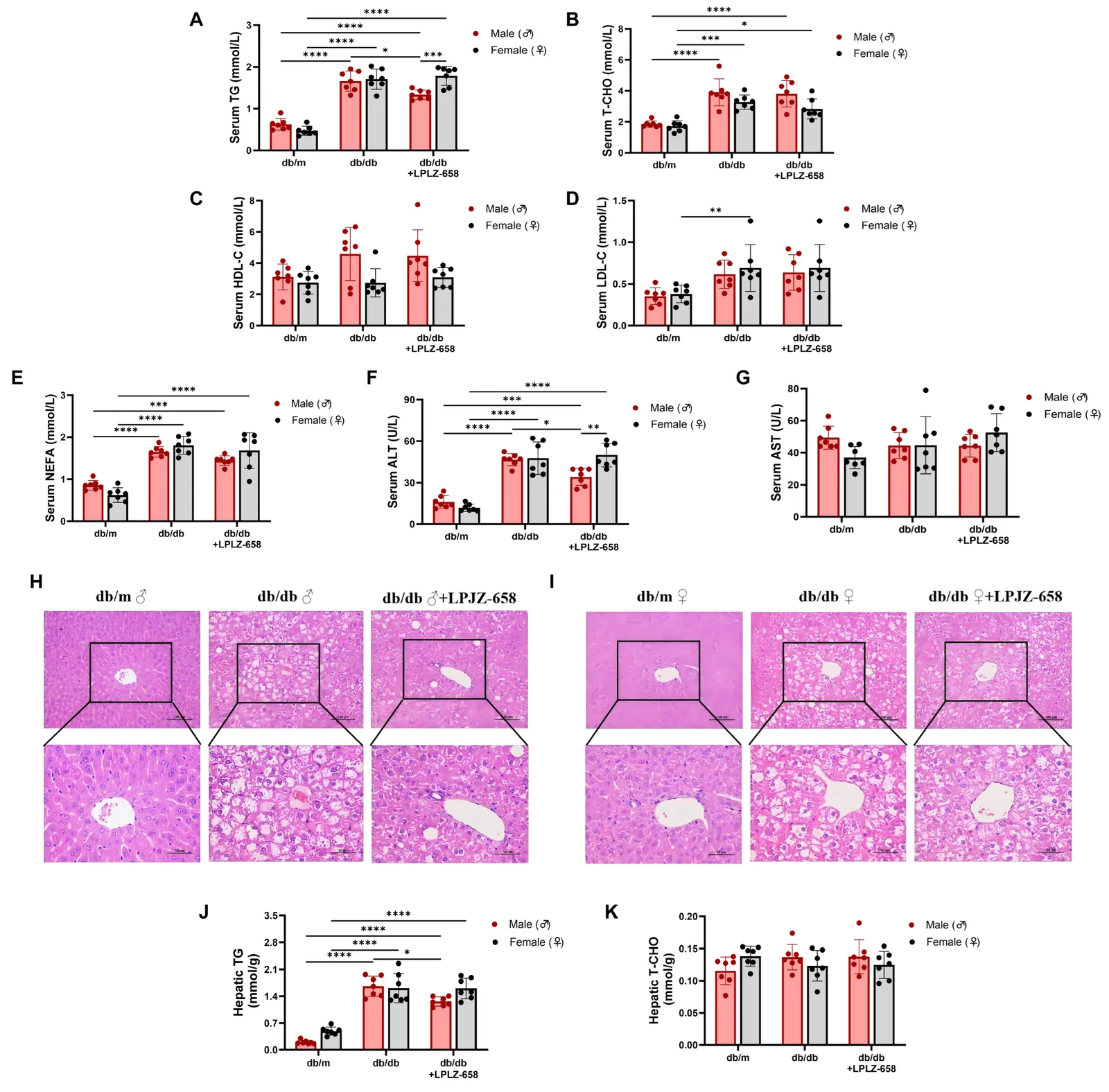
LPJZ-658 improves the serum lipid profile, alleviates liver injury, and attenuates hepatic steatosis in male db/db mice. Serum levels of (**A**) triglyceride (TG), (**B**) total cholesterol (T-CHO), (**C**) high-density lipoprotein cholesterol (HDL-C), (**D**) low-density lipoprotein cholesterol (LDL-C) and (**E**) non-esterified fatty acid (NEFA). Serum (**F**) aspartate aminotransferase (AST) and (**G**) alanine aminotransferase (ALT) levels. Representative H&E staining of liver tissue from (**H**) male mice and (**I**) female mice. Hepatic (**J**) TG and (**K**) T-CHO contents. All data are presented as the mean ± SEM (*n* = 7 for each group); * *p* < 0.05, ** *p* < 0.01, *** *p* < 0.001, and **** *p* < 0.0001. ♂, male; ♀, female.

We further examined the effect of LPJZ-658 on pancreatic tissue damage in db/db mice. As shown in Figure 3E, the pancreatic histology of both female and male mice in the db/m group was normal, with an orderly arrangement of pancreatic islet cells and distinct edges of islets and outer tissues. However, significant pancreatic damage was observed in both female and male db/db mice, which was characterized by unclear edges of islets (with blurred boundaries from surrounding tissues), atrophic islets accompanied by inflammatory cell infiltration, and a reduced number of islet cells with a disordered arrangement (blue arrows). Compared with the db/db group, only the male db/db + LPJZ-658 group exhibited significant recovery of the pancreatic structure, as evidenced by clear boundaries between islets and surrounding tissues and reduced inflammatory cell infiltration (blue arrows); no obvious improvement was observed in female db/db + LPJZ-658 mice.

### 3.3. Effects of LPJZ-658 on the Serum Lipid Profile Parameters and Liver Injury

As shown in Figure 3A–E, serum levels of TG, T-CHO, and NEFA were markedly elevated in both male and female db/db mice compared with db/m mice. Nevertheless, LPJZ-658 treatment significantly reduced serum TG levels in male db/db mice only, with no significant effects on serum T-CHO or NEFA in either sex. It is worth noting that serum HDL-C levels did not show significant changes in either male or female db/db mice. In contrast, serum LDL-C levels were significantly elevated in female db/db mice only, and treatment with LPJZ-658 did not alter serum HDL-C or LDL-C levels in either sex. As shown in Figure 3F,G, serum AST levels were comparable between db/db mice (both male and female) and db/m mice, whereas ALT levels were significantly elevated in both male and female db/db mice. Importantly, LPJZ-658 treatment significantly reversed the elevated serum ALT levels in male db/db mice only. Notably, for serum TG and ALT levels, the sex × treatment interaction and the main effect of treatment were significant. In contrast, for serum T-CHO, LDL-C, and NEFA levels, the sex × treatment interaction was not significant, while the main effect of the LPJZ-658 treatment was significant.

To further evaluate hepatic lipid accumulation, a liver histopathological examination was conducted (Figure 3H,I). H&E staining revealed increased lipid droplets in both male and female db/db mice, whereas this accumulation was attenuated by LPJZ-658 in male db/db mice only. Consistent with the histopathological observations, quantitative analysis demonstrated that hepatic TG content was significantly increased in both male and female db/db mice compared with db/m mice, and LPJZ-658 treatment reduced hepatic TG exclusively in male db/db mice. However, no significant differences in hepatic T-CHO levels were detected among any of the groups. For hepatic TG content, the sex × treatment interaction was not significant, while the main effects of LPJZ-658 treatment and sex were significant.

### 3.4. Sex-Dependent Effects of LPJZ-658 on Gut Microbiota Diversity

**Figure 4:**
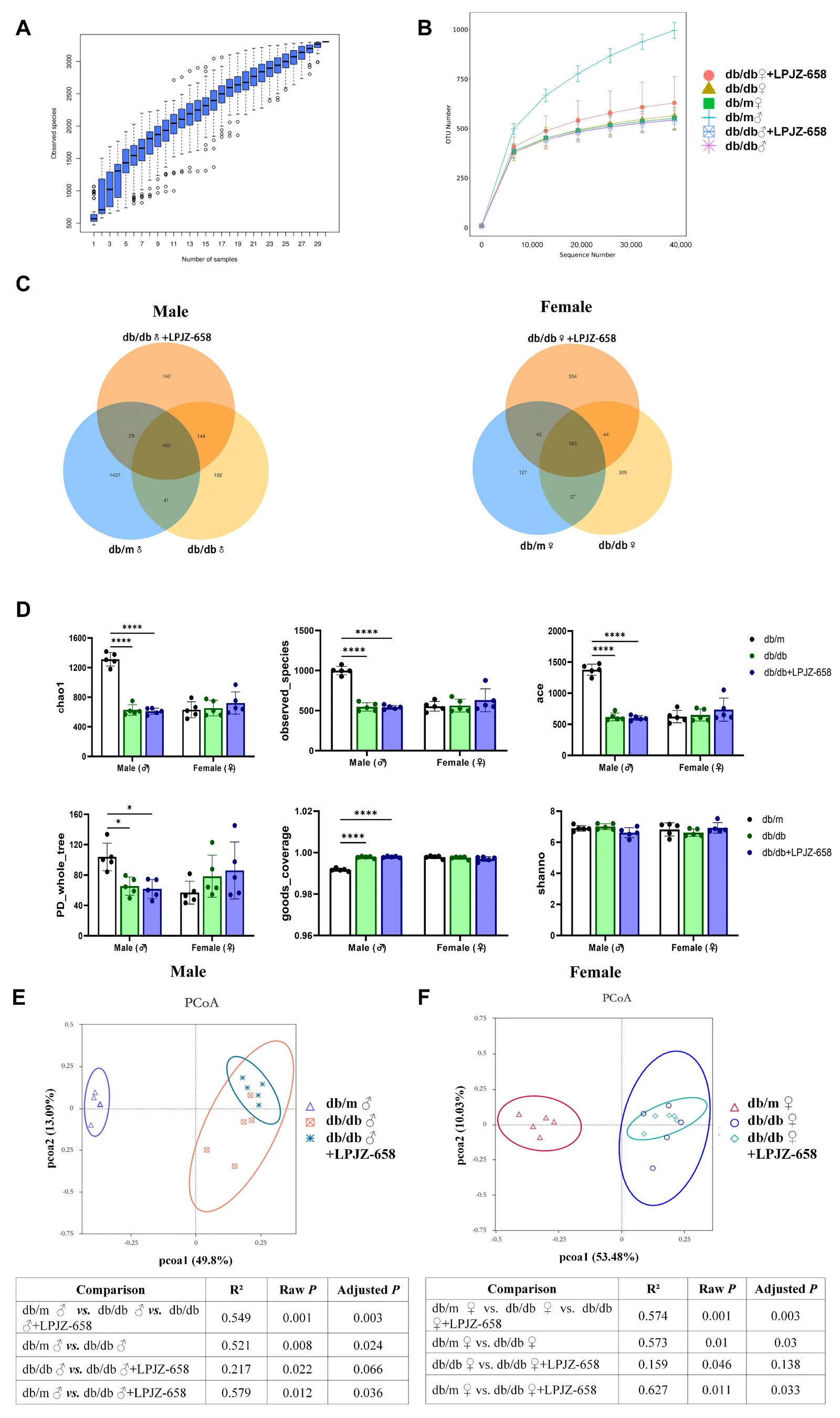
LPJZ-658 improves gut microbial diversity and community structure in a sex-dependent manner. (**A**) Species accumulation boxplot and (**B**) rarefaction curves of cecal content samples. (**C**) Venn diagram of the male (**left**) and female (**right**) mice. (**D**) Alpha diversity indices of the male and female mice. PCoA analyses of the (**E**) male and (**F**) female mice, with pairwise PERMANOVA results shown in the tables below. All data are presented as the mean ± SEM (*n* = 5 for each group); * *p* < 0.05 and **** *p* < 0.0001. ♂, male; ♀, female.

As shown in Figure 4A,B, both the species accumulation box plot and the rarefaction curve gradually flattened, indicating that the current sample size and sequencing depth met the requirements for subsequent experimental analysis. Additionally, the Good’s coverage index for all samples exceeded 95%, further verifying that the sequencing depth was sufficient to represent the complete microbial community structure, thus ensuring the reliability of subsequent analysis results. Venn diagram analysis was performed to clarify the taxonomic differences in gut microbiota between sexes (Figure 4C). Compared with female mice, male db/db and db/db + LPJZ-658 mice exhibited a significant reduction in gut microbial species diversity. Furthermore, the number of shared OTUs among the male groups was lower than that among the female groups, suggesting a decreasing trend in gut microbial richness in male mice. Additionally, a significant sex × treatment interaction for α-diversity indices was detected using two-way ANOVA, demonstrating sex-dependent microbial responses to LPJZ-658 intervention. The α-diversity analysis results (Figure 4D) showed that in male mice, the Chao1, Ace, and Observed_species indices, and PD_whole_tree indices of the gut microbiota in the db/db and db/db + LPJZ-658 groups were significantly lower than those in the db/m group, while the goods_coverage index was significantly higher than that in the db/m group. Notably, no significant differences in α-diversity indices were observed among the female groups. These findings indicate that both total microbial abundance and diversity were significantly reduced in male db/db and db/db + LPJZ-658 mice, whereas no such reduction was observed in female mice.

Principal coordinates analysis (PCoA) results (Figure 4E,F) revealed distinct separation of gut microbiota profiles between the db/m group and the db/db groups in both sexes. Because the sex × treatment interaction was significant, we performed PERMANOVA separately for male and female mice. In male mice (Figure 4E), PERMANOVA revealed significant differences between db/m and db/db and between db/m and db/db + LPJZ-658. However, the difference between db/db and db/db + LPJZ-658 was not significant after adjustment, indicating that LPJZ-658 treatment did not significantly shift the gut microbiota composition of male db/db mice, although a marginal trend toward separation was observed. In female mice (Figure 4F), PERMANOVA revealed significant differences only between db/m and db/db mice and between db/m and db/db + LPJZ-658 mice, whereas the microbial distributions of the db/db and db/db + LPJZ-658 groups were not significantly different, indicating that LPJZ-658 treatment did not alter the gut microbiota composition of female db/db mice. Together, these findings demonstrate sex-dependent differences in gut microbiota composition between db/m and db/db mice, and LPJZ-658 treatment did not significantly restore the gut microbiota composition toward that of the db/m group in either sex, although a trend toward restoration was observed in male db/db mice. Together, these findings demonstrate sex-dependent differences in gut microbiota composition between db/m and db/db mice, and only male db/db mice treated with LPJZ-658 exhibited a trend toward restoring gut microbiota composition toward that of the db/m group.

### 3.5. Sex-Dependent Effects of LPJZ-658 on Gut Microbiota Composition

**Figure 5:**
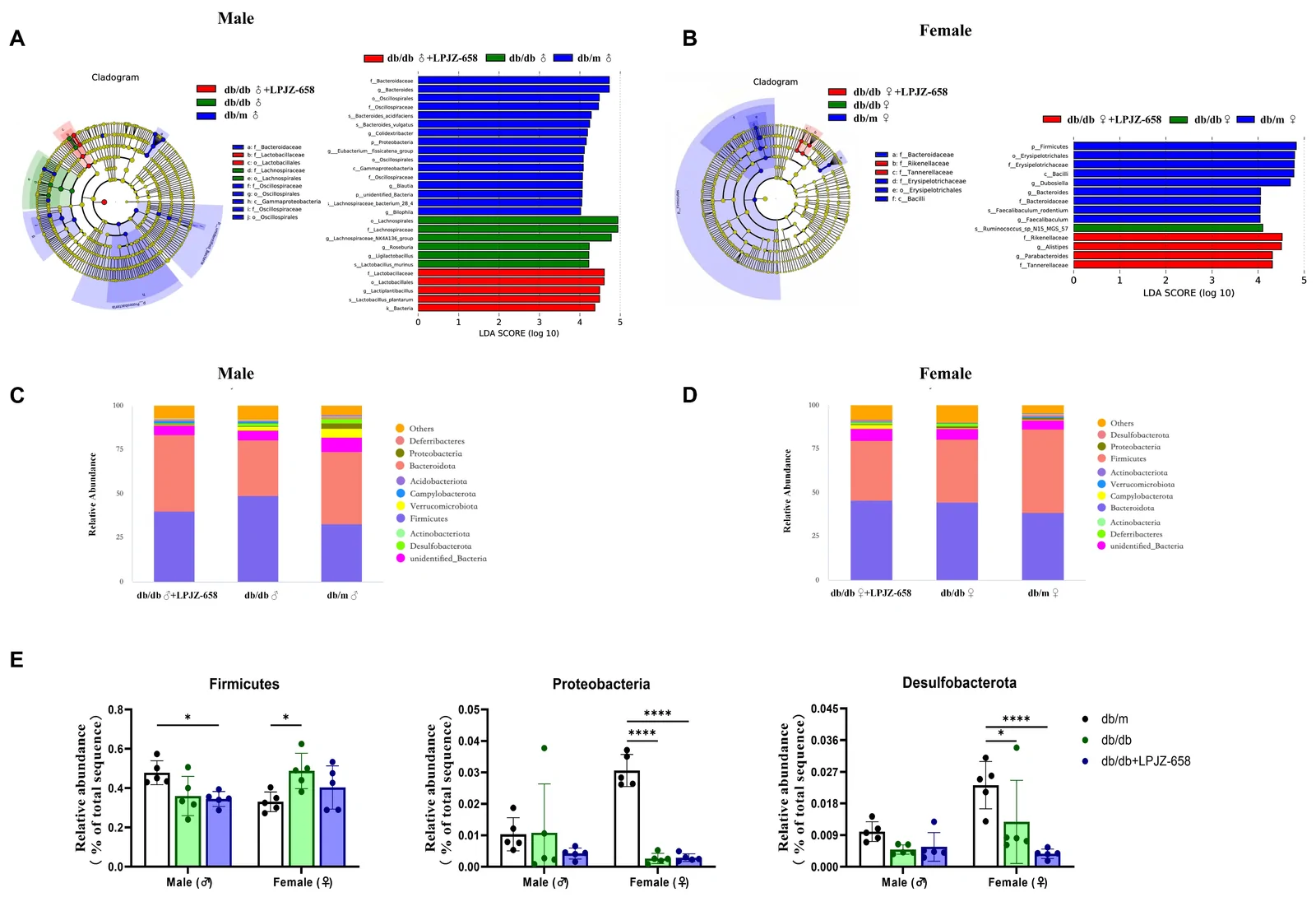
LPJZ-658 differentially modulates the composition of the gut microbiota at the phylum level in db/db mice. Linear discriminant analysis effect size (LEfSe) analysis (scores > 4) of (**A**) male and (**B**) female mice. Relative abundance of gut microbiota at the phylum level in (**C**) male and (**D**) female mice. (**E**) Relative abundance of differential phyla in male and female mice. All data are presented as the mean ± SEM (*n* = 5 for each group); * *p* < 0.05 and **** *p* < 0.0001. ♂, male; ♀, female.

Linear discriminant analysis effect size (LEfSe) analysis (LDA score  >  4, *p*  <  0.05) was used to identify the indicator microbiota taxa (biomarkers) among groups. As shown in Figure 5A, a total of 27 bacterial genera were detected among the three groups in male mice. Similarly, 14 bacterial genera were identified among the three groups in female mice (Figure 5B). To further explore the compositional characteristics of gut microbiota, we analyzed the dominant taxa at the phylum and genus levels. At the phylum level (top 10 dominant phyla), Firmicutes and Bacteroidetes were the primary dominant phyla in both sexes, collectively accounting for more than 80% of the total microbial abundance (Figure 5C,D). Additionally, two-way ANOVA revealed a significant sex × treatment interaction for Firmicutes, Proteobacteria, and Desulfobacterota (Figure 5E), while stratified analysis showed that in male mice, the abundance of Firmicutes in db/db + LPJZ-658 mice was significantly reduced compared with that in db/m mice. In female db/db mice, a significant increase in Firmicutes abundance was seen compared with db/m mice, while no significant change was observed in db/db + LPJZ-658 mice. Compared with db/m mice, both the db/db and db/db + LPJZ-658 mice displayed significantly decreased Proteobacteria and Desulfobacterota abundance.

**Figure 6:**
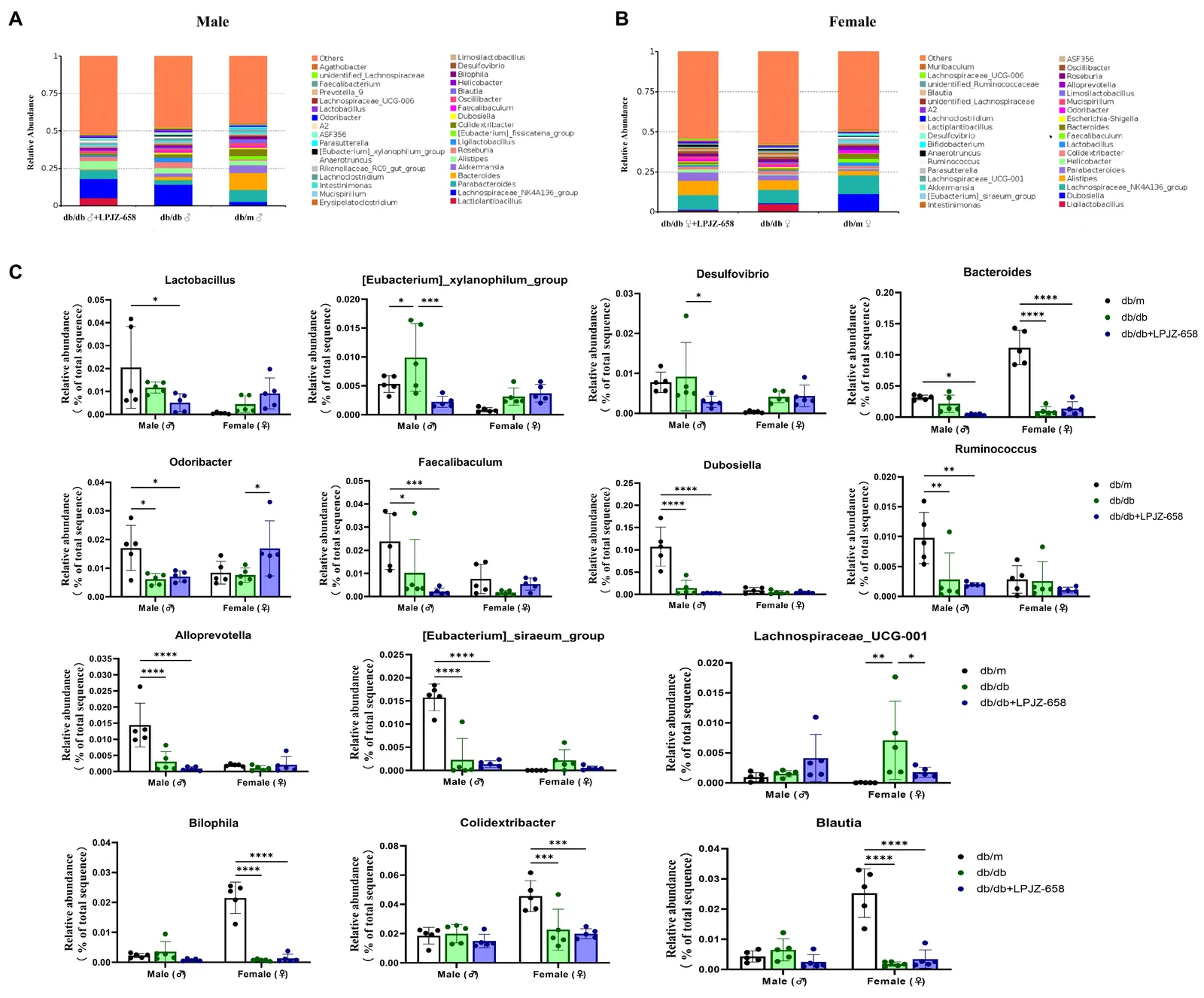
LPJZ-658 differentially modulates the composition of the gut microbiota at the genus level in db/db mice. Relative abundance of gut microbiota at the genus level in (**A**) male and (**B**) female mice. (**C**) Relative abundance of differential genera in male and female mice. All data are presented as the mean ± SEM (*n* = 5 for each group); * *p* < 0.05, ** *p* < 0.01, *** *p* < 0.001, and **** *p* < 0.0001. ♂, male; ♀, female.

At the genus level (top 35 dominant genera), the major shared dominant genera across both sexes included Ruminococcus, Roseburia, Rikenellaceae_RC9_gut_group, Parasutterella, and Parabacteroides (Figure 6A,B), and two-way ANOVA identified significant sex × treatment interactions for multiple genera (Figure 6C). In male mice, the abundance of [Eubacterium]_xylanophilum_group was markedly elevated in db/db mice relative to db/m controls, and this elevation was reversed by LPJZ-658 supplementation. Compared with db/db mice, db/db + LPJZ-658 mice exhibited significantly lower abundance of Desulfovibrio. Furthermore, reduced abundances of Lactobacillus and Bacteroides were observed in db/db + LPJZ-658 mice compared with db/m mice. Several genera, including Odoribacter, Faecalibaculum, Dubosiella, Ruminococcus, Alloprevotella, and [Eubacterium]_siraeum_group, were consistently depleted in both the db/db and db/db + LPJZ-658 groups compared with db/m mice. In female mice, Bacteroides abundance was significantly decreased in both the db/db and db/db + LPJZ-658 groups relative to db/m controls. Following LPJZ-658 intervention, Odoribacter abundance was significantly increased in db/db + LPJZ-658 mice compared with db/db mice. In addition, Lachnospiraceae_UCG-001 was enriched in db/db female mice relative to db/m mice, and such enrichment was abrogated upon LPJZ-658 treatment. Furthermore, significantly reduced abundances of Bilophila, Colidexteibacter, and Blautia were observed in both the db/db and db/db + LPJZ-658 groups compared with db/m mice. Collectively, these findings demonstrate that LPJZ-658 reshaped the gut microbiome of diabetic mice in a sexually dimorphic manner, with divergent regulatory effects on specific gut bacterial taxa between male and female hosts.

### 3.6. Sex-Dependent Effects of LPJZ-658 on KEGG Pathways

**Figure 7:**
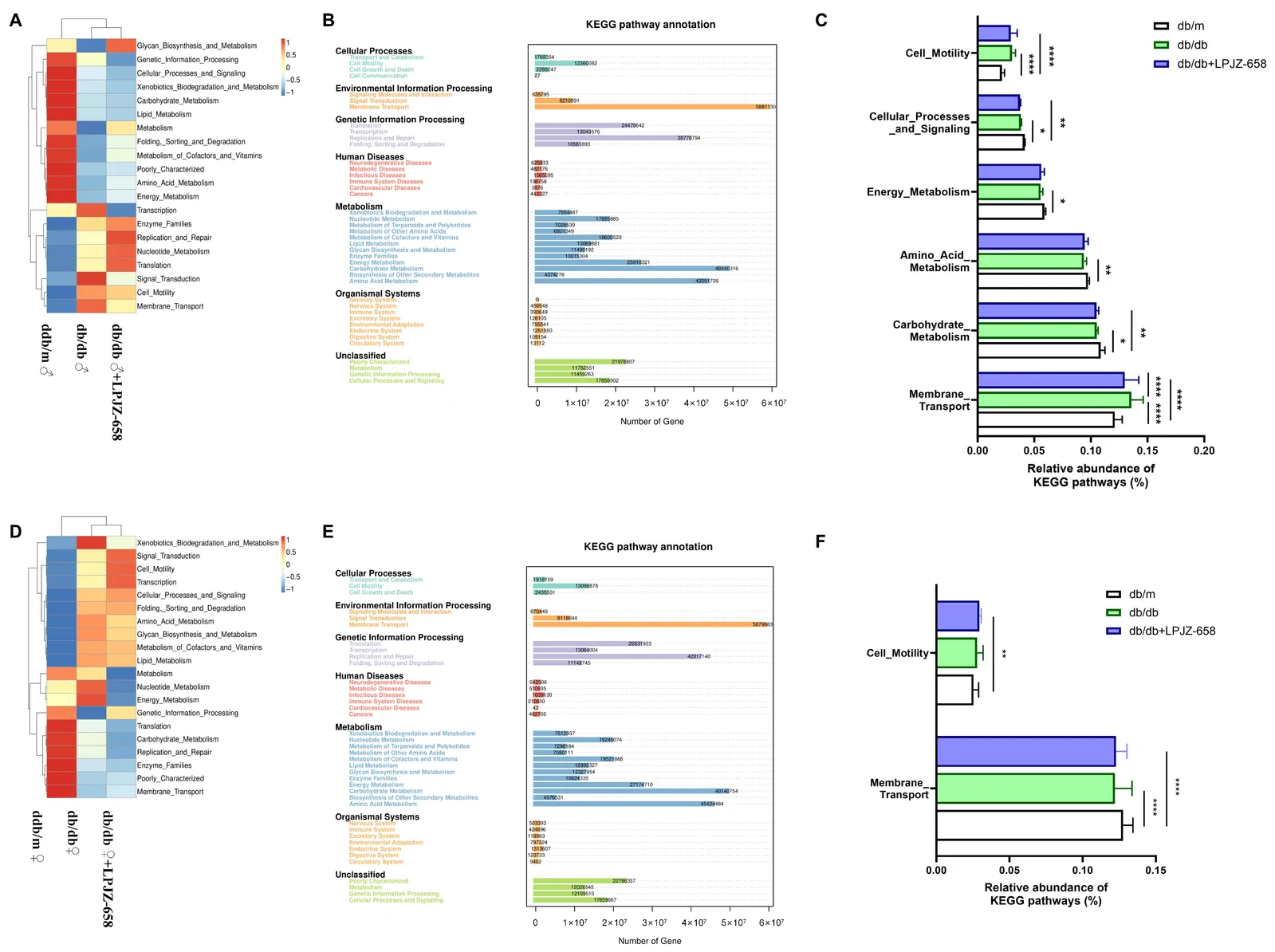
LPJZ-658 modulates gut microbiota-related functional metabolic pathways in db/db mice. (**A**) Heatmap of species abundance clustering of male mice. (**B**) Kyoto Encyclopedia of Genes and Genomes (KEGG) functional pathway annotation of the male mice. (**C**) Relative abundance of differentially abundant KEGG pathways in male mice. (**D**) Heatmap of species abundance clustering of female mice. (**E**) Kyoto Encyclopedia of Genes and Genomes (KEGG) functional pathway annotation of the female mice. (**F**) Relative abundance of differentially abundant KEGG pathways in female mice. All data are presented as the mean ± SEM; * *p* < 0.05, ** *p* < 0.01, and **** *p* < 0.0001. ♂, male; ♀, female.

Given that the most pronounced anti-diabetic effects of LPJZ-658 were observed in male db/db mice, and considering the significant sex × treatment interaction in gut microbiota composition, we performed PICRUSt analysis and KEGG pathway annotation separately for male and female mice. In male mice (Figure 7A–C), the heatmap revealed a clear separation of functional pathways between the db/m and db/db groups, indicating that diabetes significantly altered the functional potential of the gut microbiota. Notably, LPJZ-658 treatment partially reversed these alterations, as the pathway profile of the db/db + LPJZ-658 group shifted toward that of the db/m group (Figure 7A). The KEGG pathway annotation confirmed that membrane transport, carbohydrate metabolism, and amino acid metabolism were among the most abundant functional categories in male mice (Figure 6B). A quantitative comparison of differentially abundant pathways (Figure 7C) showed that LPJZ-658 treatment significantly modulated multiple pathways in male mice. Specifically, Membrane_Transport was significantly elevated in db/db mice compared with db/m mice, and LPJZ-658 treatment significantly reduced its abundance toward the db/m level, indicating a reversal of the diabetes-induced increase. Amino_Acid_Metabolism and Energy_Metabolism were significantly lower in db/db mice compared with db/m mice. Cell_Motility was significantly elevated in both the db/db and db/db + LPJZ-658 groups compared with db/m mice. In contrast, Carbohydrate_Metabolism and Cellular_Processes_and_Signaling were significantly lower in both the db/db and db/db + LPJZ-658 groups compared with db/m mice.

For female mice (Figure 7D–F), the heatmap showed that the functional pathway profiles of the db/db and db/db + LPJZ-658 groups were highly similar and clustered together while being clearly separated from the db/m group (Figure 7D). The KEGG pathway annotation revealed a similar overall functional category distribution to that of males (Figure 7E). However, quantitative analysis of differentially abundant pathways (Figure 7F) identified only two pathways with significant differences among groups: Cell_Motility and Membrane_Transport. Specifically, Cell_Motility was significantly higher in db/db + LPJZ-658 mice compared with db/m mice, while Membrane_Transport was significantly lower in both the db/db and db/db + LPJZ-658 groups compared with db/m mice. These results demonstrate that the modulation of gut microbial functional potential by LPJZ-658 is sex-dependent, with distinct pathway responses observed between male and female mice.

## 4. Discussion

This study systematically investigated the sex-dependent anti-diabetic effects of *L. plantarum* LPJZ-658 in db/db mice. We applied two-way ANOVA to test for sex × treatment interactions across all parameters and, importantly, implemented stratified analyses for variables with significant interactions. Our results revealed a striking dissociation: while most metabolic and histological parameters showed no significant sex × treatment interaction, gut microbiota α-diversity, as well as multiple phyla and genera, exhibited significant interactions. These findings demonstrate that LPJZ-658 modulates host metabolism and gut microbiota through partially distinct mechanisms, with the gut microbiota response being more strongly sex-dependent than the overall metabolic response. These findings highlight the critical role that sex plays in modulating probiotic efficacy and provide new insights into the development of personalized treatment strategies for T2DM.

Consistent with the conclusions of multiple studies indicating that *L. plantarum* strains exhibit anti-diabetic potential [27], treatment with LPJZ-658 effectively reduced fasting serum glucose and serum insulin levels in male db/db mice, accompanied by improvements in pancreatic histology, as evidenced by islet recovery and reduced inflammatory infiltration. The protective effect on pancreatic tissue is consistent with previous studies indicating that *L. plantarum* supplementation alleviates islet endoplasmic reticulum (ER) stress, inhibits β-cell apoptosis, and promotes insulin secretion [28,29,30]. However, it is noteworthy that these improvements were observed only in male mice, whereas female db/db mice did not respond to LPJZ-658 treatment. This sex-specific pattern is consistent with a recent study demonstrating that *L. plantarum* AS21 significantly ameliorated glucose intolerance in aged male mice but exerted minimal effects on metabolically stable females, an effect linked to sex-specific remodeling of mucosa-associated microbial communities and restoration of microbiota-derived citrulline in males [21]. Another study reported that probiotic benefits for blood sugar may differ by sex, with older male mice showing greater improvement than females [31]. These findings collectively support the emerging concept that probiotic efficacy in metabolic diseases is sex-dependent.

The sex differences in the efficacy of LPJZ-658 were evident not only in glucose homeostasis but also in multiple metabolic parameters. Food intake was significantly reduced in male db/db mice throughout the four-week LPJZ-658 intervention, whereas in female db/db mice, it was reduced only at Weeks 2 and 4. This sex-specific pattern of food intake reduction may partly explain why metabolic improvements were observed predominantly in males. Furthermore, male db/db mice treated with LPJZ-658 exhibited significant reductions in liver/body weight ratio, hepatic triglyceride accumulation, and serum ALT levels, indicating improvements in both lipid metabolism and hepatic damage. These findings are consistent with our previous studies, which demonstrated that LPJZ-658 alleviates non-alcoholic fatty liver disease by inhibiting lipid accumulation and regulating bile acid metabolism [25,26]. In contrast, although female db/db mice showed similar metabolic disorders, no improvement in these parameters was observed following LPJZ-658 treatment. It is important to note that the sex × treatment interaction did not reach statistical significance for most metabolic parameters, suggesting that LPJZ-658 exerts a general beneficial effect on glucose and lipid metabolism, but the magnitude of this effect is greater in males. This may be attributed to the relatively small sample size and the inherent variability of metabolic parameters, which limited the power to detect a significant interaction. Nevertheless, the consistent pattern of male-predominant benefits across multiple independent parameters strongly supports a sex-dependent response.

The potential mechanisms driving this sex-dependent response may involve complex interactions among sex hormones, gut microbiota composition, and host metabolic pathways. Studies have shown that sex steroids (particularly estrogen and testosterone) play a key role in modulating gut microbiota composition and host metabolism [32,33,34]. Estrogen has been shown to modulate the gut microbiota by promoting the growth of beneficial bacterial genera such as Lactobacillus and Bifidobacterium, reducing inflammation, and improving glucose metabolism [35]. Estrogen receptor α activation can decrease the abundance of Firmicutes and increase that of Bacteroidetes, restoring the gut microbiome to non-obese abundances [36]. Female db/db mice retain endogenous estrogen signaling despite having diabetes. This signaling is known to protect against metabolic dysfunction through various mechanisms, including enhanced insulin sensitivity, reduced hepatic gluconeogenesis, and improved lipid metabolism [37]. In female mice, the therapeutic effects of LPJZ-658 may be limited due to estrogen-mediated metabolic protection. Conversely, male mice, which lack such estrogen-mediated protection and exhibit markedly impaired metabolism, may be more responsive to probiotic supplementation. This explanation is supported by human studies: pre-menopausal women have a lower type 2 diabetes incidence than men, and women catch up with men in incidence after estrogen levels decline [38,39]; furthermore, evidence suggests that testosterone has opposite effects on glucose metabolism in men and women [40,41]. Moreover, androgens can impair glucose homeostasis partly through gut microbiota-dependent mechanisms, and depleting androgen levels via castration changes the gut microbiome of male mice to be more similar to that of females and improves glucose metabolism [31].

To date, substantial evidence of gut microbiota dysbiosis has been found in individuals with T2DM [42,43,44,45] and animal models [46,47]. In this study, our gut microbiota analysis revealed significant sex-specific differences in both baseline microbial composition and the response to LPJZ-658 treatment. Our findings revealed that, compared to db/m mice, male db/db mice exhibited significantly reduced gut microbial α-diversity. This is consistent with the results of many clinical studies, which have shown that patients with prediabetes (preDM) and newly diagnosed diabetes (newDM) exhibit lower gut microbial diversity [44,48,49]. However, it is noteworthy that such a reduction was not observed in female db/db mice. This sex-specific pattern of α-diversity reduction is consistent with previous reports demonstrating that sex is a major determinant of gut microbial community structure [35]. Furthermore, PCoA confirmed that LPJZ-658 treatment induced a marked shift in gut microbiota composition toward that of db/m mice only in male mice. This observation is consistent with a growing evidence base indicating that probiotic interventions exhibit sex-specific effects on gut microbiota composition [50,51,52].

We then conducted analyses at the phylum and genus levels, further revealing sex-specific gut microbial characteristics associated with the efficacy of LPJZ-658. At the phylum level, the Firmicutes and Bacteroidetes phyla dominated the gut microbiota in all groups, consistent with the typical structure of mammalian gut microbiota [53,54,55]. Two-way ANOVA revealed a significant sex × treatment interaction for Firmicutes, Proteobacteria, and Desulfobacterota. In male mice, LPJZ-658 significantly reduced the relative abundance of Firmicutes compared with db/db mice. This is worth noting because Firmicutes are associated with increased dietary energy intake and enhanced lipid synthesis [56,57]. Therefore, the reduction in Firmicutes abundance in male mice treated with LPJZ-658 is likely associated with the observed improvements in body weight, hepatic steatosis, and insulin resistance. In contrast, the abundance of the Firmicutes phylum in female db/db mice was significantly increased compared to that in the db/m control group, and LPJZ-658 treatment did not reverse this increase, providing a potential microbiological basis for the lack of metabolic improvement in female mice. This is consistent with a study showing that estrogen receptor α activation decreased the abundance of Firmicutes and increased that of Bacteroidetes [36], suggesting that the Firmicutes-lowering effect of LPJZ-658 in males may partially compensate for the lack of estrogen-mediated Firmicutes regulation.

Another significant sex difference was observed in Desulfobacterota and Proteobacteria. Desulfobacterota produce hydrogen sulfide (H2S), which may have anti-inflammatory effects at low concentrations but can impair intestinal barrier function and promote low-grade systemic inflammation at high concentrations [58,59]. In male mice, there were no significant changes in the abundance of Desulfovolae and Proteobacteria among the groups. In female mice, however, both the db/db and db/db + LPJZ-658 groups showed significantly decreased abundances of Desulfobacterota and Proteobacteria compared with db/m mice, suggesting that LPJZ-658 exerts different effects on these microbial communities in mice of different sexes. These phylum-level differences further support the notion that the gut microbiota response to LPJZ-658 is sex-dependent. At the genus level, two-way ANOVA revealed significant sex × treatment interactions for multiple genera, including Lactobacillus, Bacteroides, and Odoribacter. In male mice, LPJZ-658 treatment significantly reduced the abundance of the [Eubacterium]_xylanophilum_group compared with db/db mice and significantly reduced Desulfovibrio abundance compared with db/db mice. Additionally, Lactobacillus and Bacteroides were significantly reduced in male db/db + LPJZ-658 mice compared with db/m mice, while Odoribacter, Faecalibaculum, Dubosiella, Ruminococcus, Alloprevotella, and the [Eubacterium]_siraeum_group were all significantly lower in both the db/db and db/db + LPJZ-658 groups compared with db/m mice. In female mice, Bacteroides was significantly reduced in both the db/db and db/db + LPJZ-658 groups compared with db/m mice. Notably, Odoribacter was significantly higher in female db/db + LPJZ-658 mice compared with db/db mice, while Lachnospiraceae_UCG-001 was significantly higher in the db/db group but significantly lower after LPJZ-658 treatment.

PICRUSt analysis of male mice further confirmed the functional significance of sex-dependent microbiome regulation. In male mice, we identified significant alterations in multiple KEGG pathways, including membrane transport, carbohydrate metabolism, amino acid metabolism, and replication and repair pathways, following treatment with LPJZ-658. These changes in pathways likely contribute to the metabolic improvements observed in male mice, consistent with the known roles of gut microbiota-derived metabolites in regulating host energy metabolism, insulin sensitivity, and inflammation [60,61], further supporting the view that microbiota remodeling is a prerequisite for probiotic efficacy. However, several pathways showed a complex pattern. Amino acid metabolism and energy metabolism were reduced in db/db mice, and LPJZ-658 treatment did not significantly restore them. Cell motility was elevated in both the db/db and db/db + LPJZ-658 groups compared with db/m mice, while carbohydrate metabolism and cellular processes and signaling were reduced in both the db/db and db/db + LPJZ-658 groups. These results indicate that LPJZ-658 selectively rectifies certain functional pathways (e.g., membrane transport) in male mice, rather than globally reversing all diabetes-induced functional alterations. In contrast, in female mice, only two pathways showed significant differences among groups. Cell motility was significantly higher in db/db + LPJZ-658 mice compared with db/m mice, while membrane transport was significantly lower in both the db/db and db/db + LPJZ-658 groups compared with db/m mice. Notably, the direction of membrane transport change in females was opposite to that observed in males, further highlighting the sex-specific nature of the gut microbiota functional response to LPJZ-658. The lack of broad functional restoration in female mice is consistent with the absence of metabolic improvement in this sex and supports the notion that the gut microbiota is a key mediator of the sex-dependent anti-diabetic effects of LPJZ-658.

In the present study, we focused on characterizing the sex-dependent anti-diabetic and gut-microbiota-modulating phenotypes of *L. plantarum* LPJZ-658 in db/db mice. Nevertheless, several limitations of this study should be acknowledged. First, the sample size was relatively modest, which may have limited the statistical power to detect small but biologically meaningful differences, particularly for parameters with non-significant interactions. Moreover, baseline disease severity could differ between male and female db/db mice. Future studies incorporating pair-fed controls and careful matching of baseline disease severity are required to validate our present observations. Second, although we observed clear sex-dependent differences in the anti-diabetic effects of LPJZ-658 and in the gut microbiota response, we did not measure circulating sex hormone levels, nor did we perform sex hormone intervention experiments (e.g., castration or hormone supplementation). Therefore, we cannot directly establish how sex hormones regulate the therapeutic effect of LPJZ-658. Third, the functional predictions were based on PICRUSt analysis rather than direct metagenomic or metabolomic profiling. Future studies integrating metagenomics and metabolomics are warranted to identify the specific microbial metabolites and metabolic pathways that are associated with the hypoglycemic protective effect of LPJZ-658 in male mice and to explain the intrinsic mechanism underlying the lack of response in female mice. Fourth, although our findings highlight the importance of considering sex in probiotic interventions, this study was conducted only in mice; human clinical trials with balanced enrollment of male and female subjects and stratified analysis of sex effects are needed before these findings can be translated to clinical practice. Finally, the development of sex-specific probiotic-assisted intervention products for type 2 diabetes remains an important direction for future research. In summary, although the present study provides strong evidence for the sex-dependent anti-diabetic effects of LPJZ-658, further studies incorporating sex hormone manipulation, multi-omics approaches, and sex-balanced clinical trials are essential to fully elucidate the underlying mechanisms and to optimize personalized probiotic therapies for T2DM.

## 5. Conclusions

In summary, this study demonstrates that *L. plantarum* LPJZ-658 exhibited significant anti-diabetic effects in male db/db mice by improving glucose and lipid metabolism; alleviating pancreatic, renal, and hepatic damage; and modulating specific gut microbial taxa and predicted metabolic pathways. Importantly, although most metabolic parameters did not show a significant sex × treatment interaction, the gut microbiota response to LPJZ-658 was strongly sex-dependent, with significant interactions observed in α-diversity and multiple taxa at the phylum and genus levels. However, female db/db mice exhibited no significant metabolic or microbiota improvements following LPJZ-658 treatment. These findings highlight the critical importance of considering sex as a key biological variable in the development and application of probiotic-based diabetes interventions. Future studies should investigate the mechanistic basis of these sex differences, particularly the roles of sex hormones and their interactions with gut microbiota, to optimize personalized probiotic therapies for T2DM.

## Figures and Tables

**Table 1:** Experimental animal grouping and LPJZ-658 intervention protocol.

Group	Sex	Mouse Strains	Treatment	Dosage	Administration Method	Time
db/m♂	Male	C57BL/Ks	PBS	100 μL/day	i.g.	4 weeks
db/db♂	Male	db/db	PBS	100 μL/day	i.g.	4 weeks
db/db♂ + LPJZ-658	Male	db/db	LPJZ-658	1 × 10^9^ CFU/ 100 μL/day	i.g.	4 weeks
db/m♀	Female	C57BL/Ks	PBS	100 μL/day	i.g.	4 weeks
db/db♀	Female	db/db	PBS	100 μL/day	i.g.	4 weeks
db/db♀ + LPJZ-658	Female	db/db	LPJZ-658	1 × 10^9^ CFU/ 100 μL/day	i.g.	4 weeks

**Table 2:** The food intake of six experimental groups during 4 weeks.

	db/m♂	db/db♂	db/db♂ + LPJZ-658	db/m♀	db/db♀	db/db♀ + LPJZ-658
Week 1	2.243 ± 0.112	4.220 ± 0.099 ^##^	2.859 ± 0.084 ^$, ***^	2.135 ± 0.113	3.537 ± 0.111 ^###, %%^	3.155 ± 0.084 ^$$^
Week 2	2.275 ± 0.120	4.214 ± 0.114 ^###^	2.944 ± 0.166 ^$, **^	2.143 ± 0.101	3.701 ± 0.112 ^###, %^	3.234 ± 0.116 ^$$,, *^
Week 3	2.335 ± 0.124	4.172 ± 0.060 ^##^	2.703 ± 0.113 ^**^	2.151 ± 0.133	3.748 ± 0.112 ^###, %^	3.303 ± 0.120 ^$$, &^
Week 4	2.399 ± 0.068	4.261 ± 0.097 ^###^	2.758 ± 0.118 ^***^	2.245 ± 0.109	3.734 ± 0.095 ^###, $$, %^	3.286 ± 0.099 ^$$$$, *, &^

Data are expressed as mean ± SEM (*n* = 7 for each group); values with different superscript symbols are significantly different (*p* < 0.05). # db/m vs. db/db of the same sex; $ db/m vs. db/db + LPJZ-658 of the same sex; * db/db vs. db/db + LPJZ-658 of the same sex; % db/db♀ vs. db/db♂; & db/db♀ + LPJZ-658 vs. db/db♂ + LPJZ-658. One superscript symbol indicates *p* < 0.05; two superscript symbols indicate *p* < 0.01; and three superscript symbols indicate *p* < 0.001.

**Table 3:** The body weight ratio of six experimental groups during 4 weeks.

	db/m♂	db/db♂	db/db♂ + LPJZ-658	db/m♀	db/db♀	db/db♀ + LPJZ-658
Week 1	98.393 ± 1.007	100.034 ± 1.336	88.773 ± 4.073 ^$$, ***^	97.639 ± 1.486	100.188 ± 1.236 ^#^	97.132 ± 4.644 ^&^
Week 2	96.479 ± 1.132	102.576 ± 2.468 ^##^	89.009 ± 4.592 ^$, ***^	97.325 ± 1.278	102.889 ± 0.955 ^###^	98.400 ± 7.038
Week 3	97.581 ± 0.761	103.071 ± 3.182 ^#^	88.234 ± 3.650 ^$$, ****^	97.738 ± 1.622	103.337 ± 3.211 ^#^	98.334 ± 8.404
Week 4	97.738 ± 0.703	104.235 ± 3.874 ^#^	87.081 ± 4.687 ^$$, ***^	97.332 ± 1.655	103.058 ± 3.772 ^#^	98.059 ± 10.238

Data are expressed as mean ± SEM (*n* = 7 for each group); values with different superscript symbols are significantly different (*p* < 0.05). # db/m vs. db/db of the same sex; $ db/m vs. db/db + LPJZ-658 of the same sex; * db/db vs. db/db + LPJZ-658 of the same sex; & db/db♀ + LPJZ-658 vs. db/db♂ + LPJZ-658. One superscript symbol indicates *p* < 0.05; two superscript symbols indicate *p* < 0.01; three superscript symbols indicate *p* < 0.001; and four superscript symbols indicate *p* < 0.0001.

## Data Availability

All data generated or analyzed during this study are included in this published article.
